# Voices Of Our Elders: Community Engaged Research With Wabanaki Tribes In Maine Addressing Dementia

**DOI:** 10.1093/geroni/igaf122.3249

**Published:** 2025-12-31

**Authors:** Ralph Cammack, Patrik Johansson

**Affiliations:** Wabanaki Public Health and Wellness, Bangor, Maine, United States; Washington State University, Everett, Washington, United States

## Abstract

Wabanaki Public Health and Wellness is a Tribally operated public health that serves the four federally recognized Tribes in the state of Maine . Wabanaki Public Health and Wellness is the principal investigator on the Wabanaki Native American Research Center for Health (Wabanaki NARCH), which is supported by the National Institutes of Health. Wabanaki NARCH aims to enhance support systems, promote cultural continuity, and improve the overall well-being of Wabanaki Elders living with dementia. In partnership with two academic institutions, Washington State University and the University of Miami, Wabanaki Public Health and Wellness, through Wabanaki NARCH, is conducting Voices of Our Elders (VOE), an epidemiologic study that seeks to better understand the prevalence of Alzheimer’s disease and Alzheimer’s disease and related dementia (AD/ADRD) and cognitive decline. VOE also seeks to assess health and lifestyle risk factors for AD/ADRD and cognitive decline among Wabanaki Elders. This presentation will offer the perspectives and lessons learned from a Tribal public health district and its academic partners on: 1. The process of developing, adapting, and Indigenizing study designs and research methodologies of an AD/ADRD study that seeks to better serve the needs and perspectives of Wabanaki Elders; 2. Community engagement strategies employed to reach Wabanaki Elders to participate in VOE; and 3. Ethical aspects and strength-based and capacity building approaches for all research partners to consider when conducting research with Tribal communities in addition to the engagement with academic institutional review boards.

